# Exposure to Secondhand Smoke and Risk of Cancer in Never Smokers: A Meta-Analysis of Epidemiologic Studies

**DOI:** 10.3390/ijerph15091981

**Published:** 2018-09-11

**Authors:** A-Sol Kim, Hae-Jin Ko, Jin-Hyun Kwon, Jong-Myung Lee

**Affiliations:** 1 Department of Family Medicine, School of Medicine, Kyungpook National University, Kyungpook National University Chilgok Hospital, Daegu 41404, Korea; deepai@knu.ac.kr; 2 Department of Family Medicine, School of Medicine, Kyungpook National University, Kyungpook National University Hospital, Daegu 41944, Korea; 3 Department of Family Medicine, Kyungpook National University Hospital, Daegu 41944, Korea; angelica1119@gmail.com; 4 Department of Internal Medicine, School of Medicine, Kyungpook National University, Kyungpook National University Hospital, Daegu 41944, Korea; jomlee@knu.ac.kr

**Keywords:** tobacco smoke pollution, secondhand smoking, passive smoking, cancer, neoplasm, meta-analysis

## Abstract

This is first meta-analysis to evaluate cancer risk associated with secondhand smoking across all cancers. A literature search was conducted for articles published before June 2014 on Pubmed, SCOPUS, Cochrane library, and CINAHL, and 40 articles on secondhand smoke and the prevalence of cancer among never smokers were selected for final analysis as per the inclusion criteria. Of the 40 articles, 27 were case-control studies and 13 were prospective cohort studies. With respect to overall cancer risk, odds ratio (OR) involving never smokers with significant exposure to secondhand smoke compared to never smokers without such exposure was 1.163 (95%CI 1.058–1.279). Subgroup meta-analyses by study design showed significant positive associations for both case-control studies and prospective cohort studies (OR 1.165, 95%CI 1.029–1.320; and OR 1.160, 95%CI 1.002–1.343, respectively). The association was stronger in the case of females (OR 1.253, 95%CI 1.142–1.374), lung cancer (OR 1.245, 95%CI 1.026–1.511), and breast cancer (OR 1.235, 95%CI 1.102–1.385). Secondhand smoking may increase the overall risk of cancer for never smokers, particularly lung and breast cancer, and especially in women. Strict implementation of smoking cessation programs should be encouraged, not only to reduce active smoking but also to limit exposure to secondhand smoke.

## 1. Introduction

Cancer is a major cause of death worldwide and has been a leading cause of death in Korea for several decades [1]. With the steady rise in the mortality and prevalence of cancer, investigations to identify the causes and treatment of cancer have been a primary concern of healthcare providers. Smoking was first identified as a cause of cancer in 1950 [2,3]. Since then, smoking has been found to play a causative role in several types of cancers, including laryngopharyngeal, esophageal, gastric, hepatic, renal, cervical, and hematologic cancers [4]. In the light of extensive research, tobacco smoking is now recognized to produce well-known carcinogens.

Cigarette smoking is widely known as a risk factor for lung cancer. A previous meta-analysis showed that people with any history of smoking had 5.5 times the risk of developing lung cancer compared to those who never smoked. Moreover, current smokers were at 8.43 times the risk of developing lung cancer when compared to those who never smoked, indicating a strong positive correlation, irrespective of the type of lung cancer or country [5]. While cigarette smoking has been indisputably established as a major risk factor for lung cancer, it is important to note that 10–15% of lung cancer patients have no history of smoking at all and that lung cancer is a leading cause of cancer death among non-smokers [6,7]. Since the publication of initial reports from Japan [8] and Greece [9] in the 1980s, secondhand smoke has been shown to increase the risk of lung cancer, and longitudinal studies have investigated the relationship between secondhand smoke and lung cancer for the last three decades [10,11].

Despite the well-known carcinogenic nature of smoking, studies on the indirect damage from exposure to secondhand smoke or, to be more exact, sidestream smoke (which comes directly from the tip of a cigarette), are relatively scarce. For example, some studies investigating the relationship between secondhand smoke and cancer have focused on determining the threshold amount of secondhand smoke that can cause cancer or elucidating the underlying biological mechanism. Additionally, sidestream smoke is known to cause cancer by exerting both genotoxic and carcinogenic effects, and indeed, the by-products of cigarette smoking, including tobacco-specific nitrosamines (TSNAs) such as 4-(methylnitrosamino)-1-(3-pyridyl)-1-butanone (NNK)—the main cause of adenocarcinoma—have been detected in the urine of non-smokers exposed to secondhand smoke [12,13]. That is, there is good biological evidence supporting secondhand smoke as a risk factor for cancer in never smokers. Animal research has shown that secondhand smoke causes lung and nasal cancer [14]. Findings of these studies prompted epidemiological investigations involving human subjects. The results of previous studies [15,16] showed the close relevance of secondhand smoke to cancer, although many factors could have biased the interpretation of the analysis. These include the various definitions of non-smokers applied in each study, and the difficulty in accurately classifying the places where nonsmokers are exposed to secondhand smoke (public spaces, workplaces, or residences) as well as the degree of exposure to secondhand smoke. Moreover, we cannot exclude the possibility that inaccurate quantitative and qualitative approaches may have led to classification errors. Additionally, the studies have their own limitations, including the inability to allow accurate control for confounding variables, such as occupation, lifestyle, and radon exposure.

Previous epidemiological studies have provided conflicting outcomes regarding whether secondhand smoking clearly increases cancer risk; however, sufficient evidence is not available to provide a firm conclusion. Additionally, there exist no published meta-analyses investigating the relationship between secondhand smoke and cancer risk across all cancers. Thus, this study is aimed at investigating the relationship between the risk of developing various cancers and exposure to secondhand smoke by systematically reviewing recent reports and conducting a meta-analysis.

## 2. Materials and Methods

### 2.1. Literature Search

A systematic literature search was conducted in databases to retrieve articles pertaining to secondhand smoke and cancer risk. We searched for articles published before June 2014 in the Pubmed, SCOPUS (which includes EMbase and ISI Web of Science), Cochrane library, and CINAHL databases using the keywords “passive smoking,” “passive smoke,” “secondhand smoking,” or “secondhand smoke” combined with “cancer.” Only articles written in English were included. The results were exported to a reference manager (EndNote) file. After excluding duplicated articles, three authors (H.J.K., A.S.K. and J.H.K.) independently reviewed the articles based on the inclusion and exclusion criteria (see below). We also searched the references of each article to identify any other relevant articles. In the case of discrepancy, another author (J.M.L.) was consulted, and the final decision was determined by mutual discussion.

### 2.2. Study Selection

The following inclusion criteria were applied to retrieve the relevant articles: (1) study on the prevalence or incidence of cancer associated with secondhand smoke, (2) primary observational study with case-control or prospective cohort design, (3) human study, (4) study on never-smokers, (5) study on any type of histologically confirmed cancer, except skin cancer or carcinoma in situ, and (6) study with clear definition of secondhand smoke or passive smoking. Articles were excluded from the analysis if they met any of the following criteria: (1) in vivo or in vitro study, (2) review articles, letters, case reports, or meta-analysis, (3) study on cancer mortality and not incidence, (4) study with unclear subject selection criteria or those including previous smokers (5) study on childhood cancer, or (6) study on the same population sample and cancer type by same author.

### 2.3. Data Extraction

For the meta-analysis, the total number of never smokers (who had never smoked more than one cigarette a day for one year, or 365 cigarettes over their lifetime) in the cancer and control groups and the number (%) or unadjusted odd ratios (OR) of subjects exposed to secondhand smoking were extracted from the selected articles. Moreover, hazard ratios (HRs) adjusted for several covariates were extracted to determine the additional main outcome, in terms of the longest and highest exposure compared to the lowest one. Data were also collected for the following parameters: cancer type, gender, country of origin, and period of exposure to secondhand smoking.

In the case of duplicated populations (Kurahashi [17] and Hanaoka [18]; Hooker [19], Alberg [20], Gallicchio [21] and Trimble [22]), although the data were from the same cohort, the data for the different cancer types and exact subjects differed among the studies. First, we performed a meta-analysis using the duplicated cohort as independent data, and then repeated the analyses after eliminating each duplicated study.

### 2.4. Quality Assessment

The Newcastle-Ottawa Scale (NOS) was used to assess the methodological quality of the observational studies, including those with the case-control and prospective cohort design. The NOS contains 8 items and is scored from 0 to 9 stars. No definite cut-off has been defined for the NOS that signifies a high-quality study. We used the mean values of the studies selected for the current study: 7.5 stars for prospective cohort studies and 6.6 stars for case-control studies. According to the mean values, >7 stars and >6 stars were used as the NOS cut-off for prospective cohort studies and for case-control studies, respectively, to indicate a high-quality study [23].

### 2.5. Statistical Analysis

Statistical analyses were performed using Comprehensive Meta-Analysis Version 2.2.064 (Biostat, Englewood, NJ, USA). We used the random-effects model using the Dersimonian and Laird method [24] to estimate the summary odds ratio (OR) and 95% confidence intervals (CI). To assess heterogeneity, we used Cochran’s Q statistical test, which tests the null hypothesis that all studies in the meta-analysis share a common effect size. A *p* value of <0.05 was considered to indicate significant heterogeneity. Moreover, Higgins *I^2^* was calculated, which allows a determination of the proportion of the observed variance that is real. *I^2^* above 50% or 75% suggests the presence of a moderate or high heterogeneity, respectively [25]. To check for publication bias, Begg’s funnel plot, Orwin’s Fail-safe method, and Duval and Tweedie’s trim and fill method were applied. Begg’s funnel plot is a scatter plot with effect size on the *X* axis and the sample size or variance of the *Y* axis. In the absence of publication bias, the obtained plot is symmetric and shaped as an inverted funnel [26]. Orwin’s Fail-safe N could reflect the intensities of the pooled estimates. The impact of bias would be trivial if the effect size would remain unchanged, after including all the missing studies according to the Fail-safe N [27]. The Trim and Fill method offers a more nuanced perspective, generating a funnel plot that includes both the observed studies and the imputed studies [25,28].

## 3. Results

### 3.1. Description of Selected Studies

**Figure 1:**
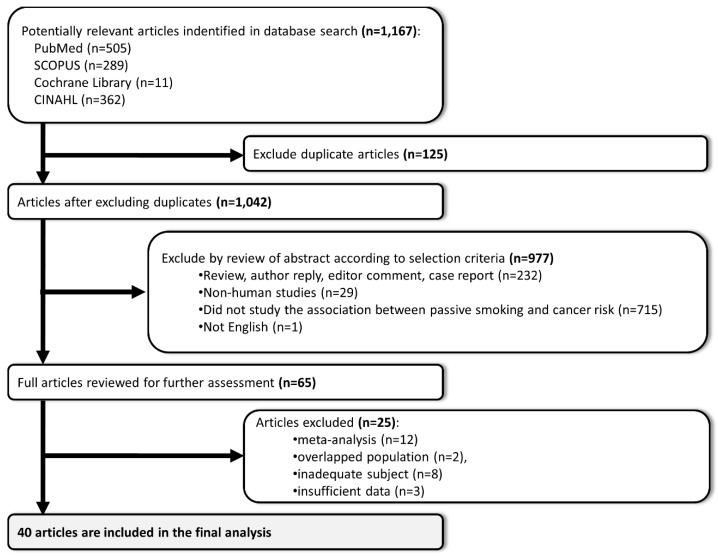
Summary of the studies selection process.

Figure 1 shows the process of study selection. The database search retrieved 1167 articles. Of them, 65 articles were fully reviewed for further assessment, and 25 articles were excluded: 12 articles because they were meta-analyses; 8 because of inadequate data or subjects; 3 because of insufficient data, and two because they included the same subjects (same cohort and same cancer). Finally, 40 articles were selected for the analysis [10,11,17,18,19,20,21,22,29,30,31,32,33,34,35,36,37,38,39,40,41,42,43,44,45,46,47,48,49,50,51,52,53,54,55,56,57,58,59,60].

The characteristics of the 40 selected studies are presented in Table 1. Twenty-seven of them were case-control studies and 13 were prospective cohort studies. As mentioned above, among the 13 prospective cohort studies, four [19,20,21,22] studies from the US (two cohorts conducted in 1963 and 1975 in two private censuses of the residents of Washington County, MD, U.S.) and two [17,18] from Japan (Japanese Public Health Center-based Prospective study) used the same cohort. As for the type of cancer, the highest frequency was noted for breast cancer (15 studies), followed by lung cancer (12 studies), bladder cancer (two studies), pancreatic cancer (two studies), and cervical, endometrial, gastric, hepatic, rectal, renal, head and neck cancers and lymphoma, (one each). Another study investigated all types of cancers. Region-wise, 32 studies were conducted in Western countries, which was four times the number of studies conducted in Asia (eight studies). Studies also showed differences in the definitions of the period of exposure to secondhand smoking, which varied from lifetime exposure and exposure during adulthood to exposure during childhood (37.5%). Most of the studies focused on the risk of cancer from exposure to secondhand smoking during adulthood. 21 studies defined never-smokers as those who had not smoked more than one cigarette a day for one year or less; 19 studies defined as those who had never smoked a cigarette.

### 3.2. Secondhand Smoking and the Risk of Cancer

**Figure 2:**
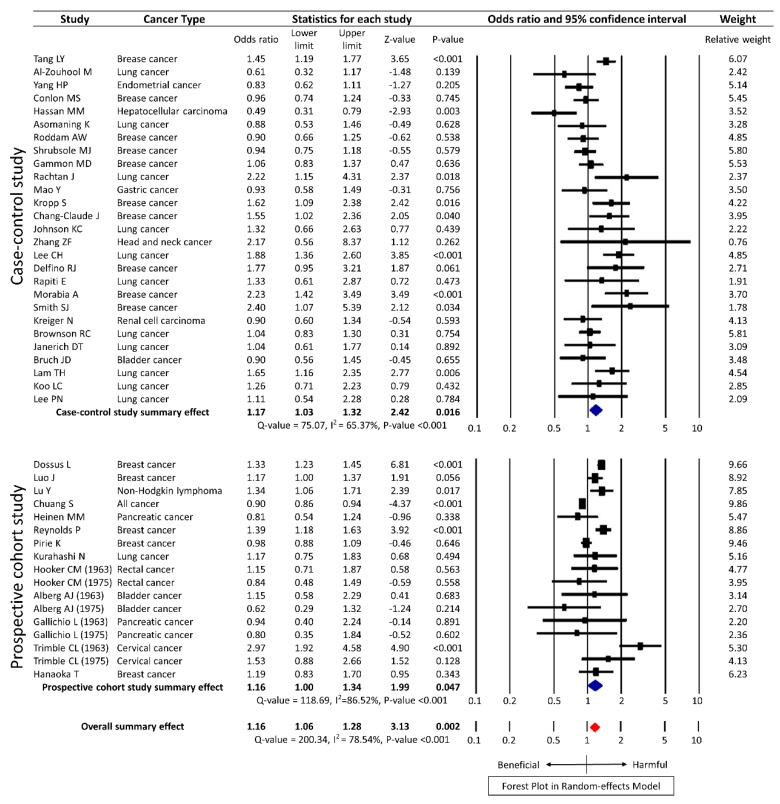
Forest plot on the association of secondhand smoke and cancer risk with unadjusted data in random-effects model.

**Figure 3:**
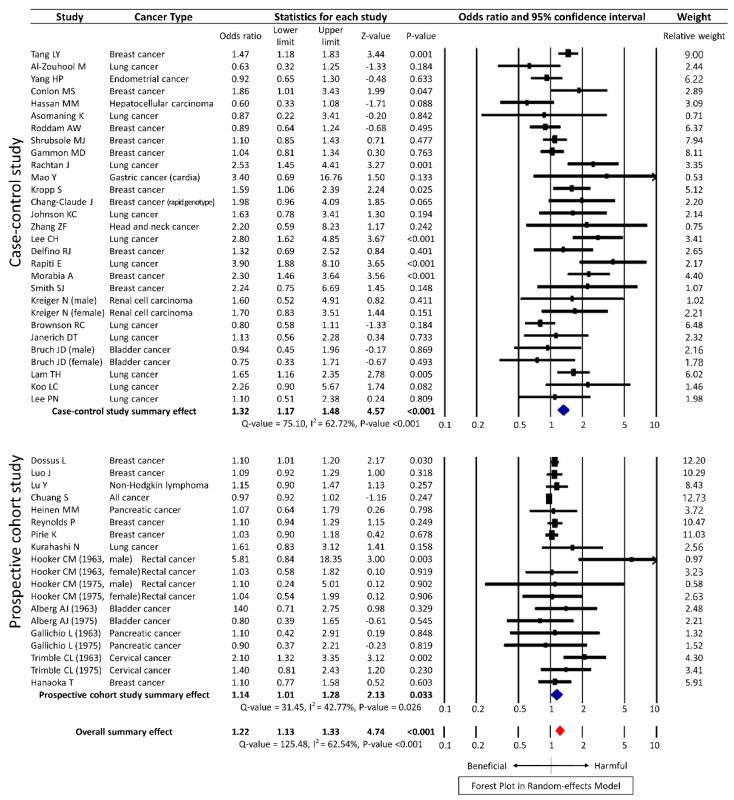
Forest plot on the association of secondhand smoke and cancer risk with fully adjusted data in random-effects model.

The meta-analysis of the unadjusted data from 40 studies using the random-effects model showed statistical significance with an OR of 1.16 (95% CI 1.06–1.28, *p* = 0.002) for overall cancer, albeit with substantial heterogeneity (*Q* = 200.34, *I^2^* = 78.54%, *p* <0.001). Among the 40 studies, 27 were case-control studies and 13 were prospective cohort studies. According to the study design, the summary effect of case-control studies was significant, with OR of 1.17 (95% CI 1.03–1.32, *p* = 0.016) and that of prospective cohort studies was 1.16 (95% CI 1.00–1.34, *p* = 0.047) (Figure 2). The meta-analysis of the adjusted data from 40 studies showed a similar result with a HR of 1.22 (95% CI 1.13–1.33, *p* < 0.001) (Figure 3).

The meta-analysis was conducted on recently published studies, which included duplicate cohorts as described in the methods section. However, meta-analysis of 36 studies (which excluded four overlapping studies by Hanaoka [18], Alberg [20], Gallicchio [21] and Trimble [22]) using the random-effects model showed that the significance of the result was maintained with OR of 1.15 (95% CI 1.04–1.26, *p* = 0.006) (heterogeneity *Q* = 173.75, *I^2^* = 79.28%, *p* < 0.001; data not shown).

### 3.3. Subgroup Analysis

Subgroup analysis was conducted to account for the different parameters. The summary effect HR for subgroup analysis of the studies on women alone was noteworthy, at 1.25 (95% CI 1.14–1.37, *p* < 0.001), contrary to the result for men with a HR of 1.59 (95% CI 0.91–2.77). Further subgroup analyses based on the association of secondhand smoking to different types of cancer yielded OR of 1.24 (95% CI 1.10–1.39, *p* < 0.001) for 15 studies on breast cancer and OR of 1.25 (95% CI 1.03–1.51, *p* = 0.026) for 12 studies on lung cancer. Analysis of two studies each on bladder cancer and pancreatic cancer did not yield any statistically significant summary estimates.

The mean NOS (quality of method) for case-control studies was 6.6 stars and was 7.5 stars for prospective cohort studies, and accordingly, 26 studies were classified as high-quality studies and 14 studies as low-quality ones. Subgroup analysis according to the confirmed methodological quality showed that the significance of the results was maintained: OR of secondhand smoking for cancer in high-quality studies was 1.15 (95% CI 1.02–1.30) and OR of secondhand smoking for cancer in low-quality studies was 1.19 (95% CI 1.02–1.39). Likewise, subgroup analysis by region (Western countries or Asia) also maintained the significance of the results (OR 1.13, 95% CI 1.01–1.25 in Western countries; OR 1.33, 95% CI 1.10–1.61 in Asia).

Subgroup analysis was performed using all exposure data, classified by the period of exposure to secondhand smoking: lifetime exposure, exposure during adulthood, and exposure during childhood. Fifteen studies were related to the risk of cancer from the exposure to secondhand smoking during childhood, and 28 studies were related to the risk from exposure during adulthood. Analysis of both subgroups did not yield any statistically significant results. Twenty-two studies examined the risk from lifetime exposure, and the OR for summary effect was statistically significant, at 1.14 (95% CI 1.02–1.27). (Table 2)

### 3.4. Publication Bias

**Figure 4:**
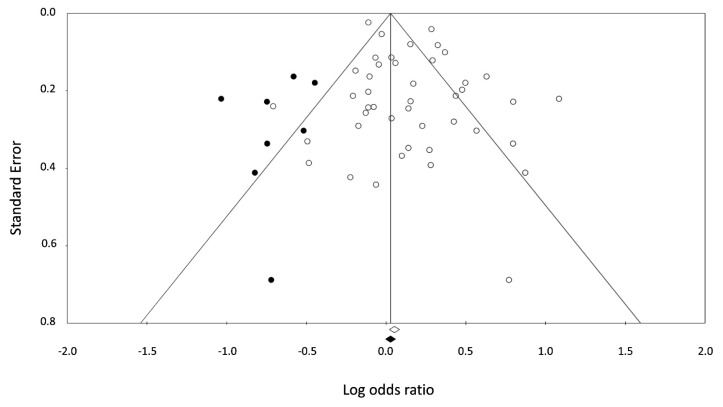
Funnel plot of standard error by log odds ratio with observed (white dot) and imputed (black dot) studies using the *Trim* and *Fill* method. Two diagonal lines represent 95% confidence limits.

Asymmetry existed in the funnel plot for the observed studies indicating the presence of publication bias (*p* = 0.785 in Begg and Mazumdar rank correlation). The OR in observed studies using Orwin’s method, however, was 1.05, suggesting that the impact of this bias was probably trivial. Similarly, after the trim and fill adjustment using Duval and Tweedie’s *Trim* and *Fill* method, the adjusted risk ratio was 1.05 (95% CI 0.95–1.15), which remained fairly unchanged. (Figure 4).

## 4. Discussion

Secondhand smoking is widely regarded as harmful for health and an environmental risk factor in terms of public health. What is intriguing, however, is the lack of a systematic meta-analysis of its effect on the increase of overall cancer risk among humans. Thus, this study was designed as a meta-analysis on 40 epidemiological studies to systematically verify whether exposure to secondhand smoking does indeed increase the risk of cancer. The findings of this study confirmed that exposure to secondhand smoking significantly increases the risk of overall cancer for never smokers. In particular, secondhand smoking significantly increases the risk of breast and lung cancer and cancers in women.

Tobacco smoking is widely known to be one of the major causes of cancer. Tobacco smoke causes exposure to approximately 7000 kinds of chemicals and 70 kinds of carcinogens such as benzopyrene, chloroethylene, *N*-nitrosamine, polycyclic aromatic hydrocarbons, aldehydes and nickel [61]. Among them, typical carcinogens, such as nitrosamine and polycyclic aromatic hydrocarbons, are metabolically activated by cytochrome P-450 enzymes, and their metabolites combine with DNA. These DNA adducts are known to cause cancer by introducing mutations in tumor suppressor genes such as *TP53* due to miscoding. In addition, they affect tumor genes such as *p53* and *KRAS*, which deregulates the process of cell growth regulation, suppresses apoptosis, and eventually causes unrestrained growth of cells [62,63].

Secondhand smoking can be classified as “mainstream smoke,” which is exhaled by a smoker after inhaling cigarette smoke, or as “sidestream smoke,” which comes directly from the tip of a cigarette. Sidestream smoke is known to contribute to 80% of the smoke in secondhand smoking [64] and since sidestream smoke burns at a lower temperature than mainstream smoke, it causes incomplete combustion, which in turn produces a thicker density of at least 17 kinds of carcinogens as compare to mainstream smoke [65,66]. Sidestream smoke is known to contain benzopyrene, one of the typical carcinogens, and *N*-nitrosodimethylalanine at concentrations that are 4.5 times and a phenomenal 100 times greater than that in mainstream smoke, respectively [66]. In fact, the application of a concentrated substance obtained from sidestream smoke to the skin of mice has been shown to more readily cause skin cancer than a concentrated substance obtained from mainstream smoke [65]. Furthermore, many animal studies have been conducted, and one study has shown that mice exposed to large amounts of secondhand smoke have an increased concentration of carcinogen-DNA adducts what are addiction products formed by covalent binding for carcinogen molecule to chemical moieties in DNA [15].

Considering these results, it is reasonable to assume that, similar to firsthand smoking, secondhand smoking is also related to the onset of cancer. A previous meta-analysis [67] showed that a non-smoking spouse has higher risk of developing lung cancer when his/her spouse is a smoker. However, another study [39] showed that the risk of breast cancer does not significantly increase for non-smoking women who are regularly exposed to cigarette smoke at home. Thus, the results of these investigations are conflict with this current study. While studies have been conducted on specific cancers such as lung and breast cancer, none have examined whether secondhand smoking generally increases the overall cancer risk. Given that smoking increases the risk of cancer for most organs of human body [60], it is crucial to verify whether this relationship is extended to secondhand smoking and overall cancer risk as well.

Therefore, this study was designed as a meta-analysis of epidemiological studies focusing on the relationship between secondhand smoking and cancer. Only studies including never-smokers were selected in order to enable the evaluation of the effect of secondhand smoking alone. Since the studies varied in design and exhibited heterogeneities with respect to various parameters, the random-effects model was chosen for the meta-analysis. Analysis of the 40 selected studies showed that secondhand smoking increased the risk for all cancers by 16%. Moreover, no significant difference was noted in terms of study design of the studies: both case–control studies and prospective cohort studies showed an increase of 16% in overall cancer risk. The finding that secondhand smoking increased the overall cancer risk by 16% is significant, considering that smoking was responsible for 20.9% of all cancer incidence in Korean adults [68] and that up to 20% of all cancer deaths can be prevented by smoking cessation worldwide [69]. Furthermore, the pooled estimate of subgroup analysis for secondhand smoking among never smokers was 1.25 in this meta-analysis, which highlights the significance of the direct and indirect effects of tobacco smoking as a risk factor for lung cancer. Prospective studies for secondhand smoking and the risk of respiratory cancers are necessary to further substantiate our findings.

Subgroup analyses were further conducted with various parameters since the studies showed heterogeneity in terms of populations and types of cancer. Many studies focused on female subjects alone, since women are more likely than men to be exposed to secondhand smoking, and such studies have shown that the risk of cancer increased by approximately 30%, which portends that secondhand smoking may have a greater effect on women. In addition, analysis based on cancer type demonstrated that secondhand smoking increased the risk of breast cancer by 24% and lung cancer by 25%. Other types of cancers showed no statistically significant results, or only single studies were conducted on some cancer types, whereby the results were insignificant. Statistical significance was maintained in the analysis based on the quality of studies and on the classification of study according to region, i.e., Western and Asian studies. Although sensitivity of individuals may differ with the type of cancer, gender of subjects, and period of exposure to secondhand smoke, it is of vital importance to disseminate that secondhand smoking raises the risk of overall cancer.

This study has several limitations. First, the included studies showed significant heterogeneities, which necessitated the use of the random effects model, which considers the average effect size as the estimated mean value of the distribution of effect sizes for heterogeneous populations. Second, this study did not account for other risk factors of cancer; this was because the adjusted factors differed across the studies (adjusted covariates of each study are shown in the Appendix A Table A1), and therefore, unadjusted data was mainly used in the current study. However, the result using adjusted data remained unchanged, and we thus considered that the effect of other risk factors to be minimal. Third, some extent of publication bias was found in this meta-analysis. However, the results after adjustment with the *Trim* and *Fill* method were similar to the existing results, thereby indicating that the bias was only marginal. Fourth, selection and recall bias may be possible in this study because it involves only observational studies. Fifth, since only never smokers were selected for this meta-analysis, there is a possibility of overestimation of the result, because breast cancer and female patients accounted for the majority of the selected study subjects. Sixth, genetic and ethnic susceptibility to cancer was not taken into consideration in this study. For these limitations, our study could not confirm definite conclusion. Further studies are warranted to investigate the relations of secondhand smoke on other cancers and ethnicity.

Despite these limitations, this meta-analysis has certain merits, including the following: extensive data on secondhand smoking were reviewed precisely by strict inclusion criteria; rather than the fixed-effects model, the random-effects model, which considers weights, was used for analysis of all the effect sizes; subgroup analyses in this study included various factors such as study design and types of cancer. Most importantly, this is the first meta-analysis to investigate the effect of secondhand smoking on overall cancer risk.

## 5. Conclusions

In conclusion, our study results indicate that secondhand smoking increases the risk of cancer, especially in the case of lung and breast cancers and women. In the light of our findings, we believe that history taking in clinical settings should include thorough inquiry regarding exposure to secondhand smoke at the patients’ workplaces and homes. In addition, the finding that secondhand smoking causes cancer in nonsmokers is important not only to the development of public health policies but also in the social and economic context, and it highlights the need for active efforts by medical professionals and government to minimize exposure of the non-smoking public to secondhand smoke.

## Figures and Tables

**Table 1:** Characteristics of selected studies on the relationship between secondhand smoking and cancer among never smokers.

Study	Country	Study Design	Number of Cases (Female %)	Number of Controls (Female %)	Cancer Type	Secondhand Smoking Exposure	Quality Assessment (Stars)
Dossus L. (2014) [29]	Europe	Prospective Cohort	78,217 (100)	26,072 (100)	Breast cancer	Lifetime *	8
Tang L.Y. (2013) [30]	China	Case-control	765 (100)	818 (100)	Breast cancer	Adulthood *	6
Al-Zoughool M. (2013) [31]	Canada	Case-control	44 (70.45)	436 (70.64)	Lung cancer	Childhood, Adulthood, Lifetime *	7
Luo J. (2011) [32]	USA	Prospective Cohort	1692 (100)	39,330 (100)	Breast cancer	Childhood, Adulthood, Lifetime *	8
Lu Y. (2011) [33]	USA	Prospective Cohort	56,015 (100)	22,991 (100)	Non-Hodgkin lymphoma	Childhood, Adulthood, Lifetime *	8
Chuang S.C. (2011) [34]	Europe	Prospective Cohort	72,091 (86.8)	33,887 (86.7)	All cancer	Childhood *	8
Yang H.P. (2010) [35]	Poland	Case-control	358 (100)	898 (100)	Endometrial cancer	Lifetime *	7
Heinen M.M. (2010) [36]	Netherland	Prospective Cohort	1029 (94.5)	310 (58.7)	Pancreatic cancer	Adulthood *^,^^‡^	8
Conlon M.S. (2010) [37]	Canada	Case-control	347 (100)	775 (100)	Breast cancer	Lifetime *	7
Reynolds P. (2009) [38]	USA	Prospective Cohort	49,468 (100)	7070 (100)	Breast cancer	Childhood, Adulthood, Lifetime *	8
Pirie K. (2008) [39]	UK	Prospective Cohort	174,819 (100)	35,828 (100)	Breast cancer	Adulthood, Lifetime *	6
Kurahashi N. (2008) [17]	Japan	Prospective Cohort	21,083 (100)	7331 (100)	Lung cancer	Adulthood *	8
Hooker C.M. (2008) [19]	USA	Prospective Cohort (1963)	7117 (86.9)	11,722 (72.9)	Rectal cancer	Adulthood *	7
	USA	Prospective Cohort (1975)	4929 (82.5)	15,245 (71.5)			
Hassan M.M. (2008) [40]	USA	Case-control	88 (53.4)	471 (52.4)	Hepatocellular carcinoma	Childhood, Adulthood, Lifetime *	7
Asomaning K. (2008) [41]	USA	Case-control	138 (59)	466 (62)	Lung cancer	Lifetime *^,‡^	6
Roddam A.W. (2007) [42]	UK	Case-control	297 (100)	310 (100)	Breast cancer	Lifetime *	8
Alberg A.J. (2007) [20]	USA	Prospective Cohort (1963)	7117 (NA)	11,722 (NA)	Bladder cancer	Adulthood *	7
	USA	Prospective Cohort (1975)	4932 (NA)	15,249 (NA)			
Gallicchio L. (2006) [21]	USA	Prospective Cohort (1963)	7117 (86.9)	11,722 (72.9)	Pancreatic cancer	Adulthood *	7
	USA	Prospective Cohort (1975)	4932 (82.5)	15,249 (71.5)			
Trimble C.L. (2005) [22]	USA	Prospective Cohort (1963)	6184 (100)	8538 (100)	Cervical cancer	Adulthood *	7
	USA	Prospective Cohort (1975)	4071 (100)	10,907 (100)			
Hanaoka T. (2005) [18]	Japan	Prospective Cohort	14,533 (100)	5660 (100)	Breast cancer	Lifetime *	8
Shrubsole M.J. (2004) [43]	China	Case-control	1013 (100)	1117 (100)	Breast cancer	Adulthood *	8
Gammon M.D. (2004) [44]	USA	Case-control	598 (100)	627 (100)	Breast cancer	Lifetime *	6
Rachtan J. (2002) [45]	Poland	Case-control	54 (100)	251 (100)	Lung cancer	Childhood *	8
Mao Y. (2002) [46]	Canada	Case-control	132 (0)	343 (0)	Gastric cancer	Lifetime *	7
Kropp S. (2002) [47]	German	Case-control	197 (100)	454 (100)	Breast cancer	Childhood, Adulthood, Lifetime *	7
Chang-Claude J. (2002) [48]	German	Case-control	174 (100)	365 (100)	Breast cancer	Childhood, Adulthood, Lifetime *	7
Johnson K.C. (2001) [11]	Canada	Case-control	71 (100)	761 (100)	Lung cancer	Childhood, Adulthood, Lifetime *	6
Zhang Z.F. (2000) [49]	USA	Case-control	26 (NA)	59 (NA)	Head and neck cancer	Lifetime *	6
Lee C.H. (2000) [10]	Taiwan	Case-control	268 (100)	445 (100)	Lung cancer	Lifetime *^,†^	7
Delfino R.J. (2000) [50]	USA	Case-control	64 (100)	147 (100)	Breast cancer	Adulthood *	5
Rapiti E. (1999) [51]	India	Case-control	58 (70.7)	123 (54.5)	Lung cancer	Childhood, Adulthood *	4
Morabia A. (1996) [52]	Switzerland	Case-control	126 (100)	620 (100)	Breast cancer	Adulthood	8
Smith S.J. (1994) [53]	UK	Case-control	204 (100)	199 (100)	Breast cancer	Childhood, Adulthood, Lifetime *	7
Kreiger N. (1993) [54]	Canada	Case-control	119 (60.5)	524 (65.8)	Renal cell carcinoma	Adulthood *	7
Brownson R.C. (1992) [55]	USA	Case-control	431 (100)	1166 (100)	Lung cancer	Childhood, Adulthood *	5
Janerich D.T. (1990) [56]	USA	Case-control	191 (NA)	191 (NA)	Lung cancer	Childhood, Adulthood, Lifetime *	7
Burch J.D. (1989) [57]	Canada	Case-control	142 (57.0)	217 (48.4)	Bladder cancer	Adulthood *^,‡^	7
Lam T.H. (1987) [58]	Hong Kong	Case-control	199 (100)	335 (100)	Lung cancer	Adulthood *	7
Koo L.C. (1987) [59]	Hong Kong	Case-control	88 (100)	137 (100)	Lung cancer	Childhood, Adulthood, Lifetime *	7
Lee P.N. (1986) [60]	UK	Case-control	47 (68.0)	96 (68.8)	Lung cancer	Adulthood *	5

* Used in main analyses. † The data of lifetime exposure was used in main analyses, because the determination of adulthood or childhood was uncertain. ‡ The data of household exposure was used in main analyses.

**Table 2:** Subgroup analysis in the random-effects model.

Variable	Studies	Estimated Effect Size (HR or OR)	95% CI	*p*-value	*I^2^* Value, %
**Sex**					
Female only	29 studies [15,16,17,20,27,28,30,31,32,33,34,35,36,37,40,41,42,43,45,47,49,50,52,53,54,55,57,58,59]	1.25	1.14–1.37	<0.001	56.14
Male only	4 studies [17,44,54,57]	1.59	0.91–2.79	0.100	44.27
**Cancer type**					
Breast cancer	15 studies [16,27,28,30,35,36,37,40,41,42,45,46,50,52,53]	1.24	1.10–1.39	<0.001	73.90
Lung cancer	12 studies [15,29,39,43,47,49,51,55,56,58,59,60]	1.25	1.03–1.51	0.026	48.20
Bladder cancer	2 studies [18,57]	0.89	0.63–1.25	0.492	0.00
Pancreatic cancer	2 studies [19,34]	0.83	0.59–1.17	0.291	0.00
Cervical cancer	1 study [20]	2.18	1.15–4.16	0.018	70.54
Endometrial cancer	1 study [33]	0.83	0.62–1.11	0.205	0.00
Gastric cancer	1 study [44]	0.93	0.58–1.49	0.756	0.00
Hepatic cancer	1 study [38]	0.49	0.31–0.79	0.003	0.00
Rectal cancer	1 study [17]	1.01	0.70–1.46	0.949	0.00
Renal cancer	1 study [54]	0.90	0.60–1.34	0.593	0.00
Head and neck cancer	1 study [48]	2.17	0.56–8.37	0.262	0.00
Lymphoma	1 study [31]	1.34	1.06–1.71	0.017	0.00
All cancer	1 study [32]	0.90	0.86–0.94	<0.001	0.00
**Methodological quality**					
High quality	26 studies [15,16,27,29,30,31,32,33,34,35,36,38,40,41,43,44,45,46,49,52,53,54,56,57,58,59]	1.15	1.02–1.30	0.023	84.00
Low quality	14 studies [17,18,19,20,28,37,39,42,47,48,50,51,55,60]	1.19	1.02–1.39	0.031	59.81
**Country**					
Western	32 studies [17,18,19,20,27,29,30,31,32,33,34,35,36,37,38,39,40,42,43,44,45,46,47,48,50,52,53,54,55,56,57,60]	1.13	1.01–1.25	0.026	79.20
Asian	8 studies [15,16,28,41,49,51,58,59]	1.33	1.10–1.61	0.003	57.24
**Period of exposure to secondhand smoking**					
Childhood	15 studies [28,30,31,32,36,38,43,45,46,47,51,53,55,56,59]	1.00	0.81–1.23	0.998	83.93
Adulthood	28 studies [15,17,18,19,20,28,29,30,31,34,36,37,38,41,45,46,47,50,51,52,53,54,55,56,57,58,59,60]	1.03	0.77–1.39	0.840	95.90
Lifetime	22 studies [16,27,29,30,31,33,35,36,37,38,39,40,42,44,45,46,47,48,49,53,56,59]	1.14	1.02–1.27	0.023	70.89

## Appendix A

**Table A1:** Adjusted covariates of each study.

Study	Adjusted Factors
Dossus L. (2014) [29]	Body mass index, educational level, hormone use, menopausal status, parity and age at first-term pregnancy, age at menarche, alcohol consumption, physical activity.
Tang L.Y. (2013) [30]	Age, marital status, long-term alcohol use, age at menarche, body mass index, parity, education, family history of breast cancer.
Al-Zoughool M. (2013) [31]	Sex, age, educational level, household income.
Luo J. (2011) [32]	Age, ethnicity, education, body mass index, physical activity, alcohol intake, parity, family history of breast cancer, hormone therapy, age at menarche, age of first live birth.
Lu Y. (2011) [33]	Age, race, alcohol intake.
Chuang S.C. (2011) [34]	Age, sex, study canter, education, alcohol intake, body mass index, physical activity, vegetable intake, fruit intake, energy intake, adulthood passive smoking.
Yang H.P. (2010) [35]	Age, study site, education, age at menarche, parity, oral contraceptives, hormone use, body mass index, menopausal status.
Heinen M.M. (2010) [36]	Age, body mass index, education.
Conlon M.S. (2010) [37]	Age.
Reynolds P. (2009) [38]	Age, race, family history of breast cancer, age at menarche, pregnancy history, lifetime duration of breast-feeding, physical activity, alcohol intake, body mass index, menopausal status, hormone use.
Pirie K. (2008) [39]	Age, region of residence, socio-economic status, age at menarche, parity, age at first birth, menopausal status, body mass index, physical activity, alcohol intake, hormone use.
Kurahashi N. (2008) [17]	Age, area, alcohol intake, family history of lung cancer, menopausal status.
Hooker C.M. (2008) [19]	Age, education, marital status.
Hassan M.M. (2008) [40]	Age, sex, race, education, marital status, area, Hepatitis C, Hepatitis B, diabetes, alcohol intake, family history of cancer.
Asomaning K. (2008) [41]	Age, sex, smoking status.
Roddam A.W. (2007) [42]	Age, region, socioeconomic status, alcohol intake, body mass index, parity, age at first birth, hormone use, family history of breast cancer, age at menarche, menopausal status.
Alberg A.J. (2007) [20]	Age, education, marital status.
Gallicchio L. (2006) [21]	Age, education, marital status.
Trimble C.L. (2005) [22]	Age, education, marital status.
Hanaoka T. (2005) [18]	Age, public health center, employment status, education level, body mass index, family history of breast cancer, past history of benign breast disease, age at menarche, number of births, menopausal status, hormone use, alcohol intake.
Shrubsole M.J. (2004) [43]	Age, education, family history of breast cancer, personal history of fibroadenoma, age at menarche, parity, age at first live birth, menopausal status, physical activity, waist-to-hip ratio.
Gammon M.D. (2004) [44]	Age, history of benign breast disease, body mass index, family history of breast cancer, history of fertility problems, number of pregnancies, menopausal status, weight in year prior to reference date.
Rachtan J. (2002) [45]	Age.
Mao Y. (2002) [46]	Age, province, education, social class, food consumption.
Kropp S. (2002) [47]	Age, alcohol intake, breastfeeding, education family history of breast cancer, menopausal status, body mass index.
Chang-Claude J. (2002) [48]	Age, number of pregnancy, breastfeeding, body mass index, alcohol intake, family history, education, menopausal status.
Johnson K.C. (2001) [11]	Age, province, education, fruit and vegetable consumption.
Zhang Z.F. (2000) [49]	Age, sex.
Lee C.H. (2000) [10]	Residential area, education, occupation, tuberculosis, cooking fuels and fume extractor.
Delfino R.J. (2000) [50]	Age, menopausal status, family history of breast cancer.
Rapiti E. (1999) [51]	Age, sex, residence, religion.
Morabia A. (1996) [52]	Age, education, body mass index, age at menarche, age at first live birth, hormone use, family history of breast cancer, history of breast biopsy.
Smith S.J. (1994) [53]	Age, region, age at menarche, nulliparity, age at first full-term pregnancy, breastfeeding, hormone use, family history of breast cancer, biopsy for benign breast disease, alcohol intake.
Kreiger N. (1993) [54]	Age, combined Quetelet index.
Brownson R.C. (1992) [55]	Age, history of previous lung disease.
Janerich D.T. (1990) [56]	Age, sex, country of residence.
Burch J.D. (1989) [57]	Age, sex, location.
Lam T.H. (1987) [58]	Age.
Koo L.C. (1987) [59]	Age, number of live births, education, years since exposure.
Lee P.N. (1986) [60]	Age, sex, hospital region, hospital ward, time of interview.
